# Substantial impact of the COVID-19 pandemic on the reported number of diagnosed chronic hepatitis C virus infections in the Netherlands, 2019–2021

**DOI:** 10.1186/s12889-023-16143-3

**Published:** 2023-06-27

**Authors:** Elisabeth M. den Boogert, Irene K. Veldhuijzen, Ellen Generaal, Maria Prins, Milan J. Sonneveld, Adriaan J. van der Meer, Paul Zantkuijl, Birgit H.B. van Benthem, Eline L.M. Op de Coul

**Affiliations:** 1 Centre for Infectious Disease Control, Epidemiology and Surveillance, National Institute for Public Health and the Environment (RIVM), Bilthoven, the Netherlands; 2 ECDC Fellowship Programme, Field Epidemiology Path (EPIET), European Centre for Disease Prevention and Control (ECDC), Stockholm, Sweden; 3 Department of Infectious Diseases, Research and Prevention, Public Health Service of Amsterdam, Amsterdam, the Netherlands; 4 Department of Infectious Diseases, Amsterdam UMC, Amsterdam Infection and Immunity (AII), University of Amsterdam, Amsterdam, the Netherlands; 5 Department of Gastroenterology & Hepatology, Erasmus MC University Medical Center, Rotterdam, the Netherlands; 6 Soa Aids Nederland, Amsterdam, the Netherlands

**Keywords:** SARS-CoV-2, Chronic Hepatitis C, Men who have sex with men, Netherlands, Epidemiology

## Abstract

**Background:**

The COVID-19 pandemic has widespread consequences for health facilities, social contacts, and health-seeking behaviour, affecting the incidence, diagnosis and reporting of other infectious diseases. We examined trends in reported chronic hepatitis C virus (HCV) infections and associated transmission routes in the Netherlands to identify the potential impact of COVID-19 on access to healthcare (testing) services.

**Methods:**

We analysed notification data of patients with chronic HCV reported to the National Notifiable Disease Surveillance System from January 2019 until December 2021 in the Netherlands. Rates of newly reported chronic cases per 100,000 population with 95% confidence intervals (CI) were calculated, and we compared proportional changes in transmission routes for chronic HCV between 2019, 2020 and 2021.

**Results:**

During the study period, a total of 1,521 chronic HCV infections were reported, 72% males, median age 52 years, and an overall rate of 8.8 (95%CI 8.4–9.2) per 100,000 population. We observed an overall decline (-41.9%) in the number of reported chronic HCV in 2020 compared to 2019, with the sharpest decline in men who have sex with men (MSM)-related transmission (-57.9% in 2020, p = 0.005).

**Conclusions:**

Reported cases of chronic HCV strongly declined during the COVID-19 pandemic when healthcare services were scaled down. Between February and June 2021, reported chronic HCV cases increased again, indicating a recovery of healthcare services. MSM showed the largest decline compared to other groups. Further research is needed to fully understand the impact of access to healthcare, health seeking behaviour, and (sexual) transmission risks of HCV during the COVID-19 pandemic.

Hepatitis C virus (HCV) is a blood borne virus that can lead to recently acquired and chronic HCV infection. Without treatment, a recently acquired infection becomes chronic in approximately 75% of the cases [1]. If untreated, chronic infection could lead to liver cirrhosis and cancer [2]. Worldwide an estimated 71 million people are living with a chronic HCV infection [3]. In the Netherlands around 23 thousand people (8,000–38,000) were estimated to have (had) chronic HCV in 2016; about 0.16% of the adult population [4]. Since 2016, direct-acting antivirals (DAA) became available for treating HCV. Treatment with DAA is highly effective [5], and sustained virological response is associated with a marked improvement in prognosis [6].

In 2016 the World Health Assembly set goals to eliminate viral hepatitis, including hepatitis C, as a public health problem, and published updated strategies in 2022 [7]. Before 2030, the aim is to reduce new infections by 90%, to reduce hepatitis related deaths by 65%, to diagnose at least 90% of people with hepatitis C, and at least 80% of those should receive appropriate treatment, compared with a 2015 baseline [8]. In 2016, a Dutch National Hepatitis Plan [9] was developed, which implemented strategies to reach these elimination goals. This included (innovative) identification of the heterogenous group of undiagnosed patients and previously diagnosed but untreated patients [10].

In 2020, SARS-CoV-2, the virus causing COVID-19, was introduced in the Netherlands and impacted society and health care systems. A variety of social distancing measures was introduced, including a lockdown from March-May 2020 and a second, less-strict, lockdown from mid-October 2020 until January 2021. From February 2021 onwards, an evening curfew, among others, was implemented. When more people became vaccinated against SARS-CoV-2, restrictions were gradually lifted from May 2021 onwards [11]. In 2021, Sonneveld et al. reported a significant decrease of 40% in chronic hepatitis C diagnoses that mirrored the COVID-19 hospital admissions [12]. They highlighted the importance to refocus on the WHO elimination goals by identifying and linking undiagnosed hepatitis patients to testing and care. Recently, van Dijk et al. estimated the effect of COVID-19 on the elimination of HCV in a mathematical model, by including an annual reduction of 42% in diagnosed (and treated) HCV patients [13]. When including this COVID-19 effect in the model, the Netherlands would still be on track to reach the elimination goals by 2030. However, the modeled COVID-19 scenarios resulted in a prediction of more cases of decompensated liver cirrhosis and hepatocellular carcinoma [13, 14].

In the Netherlands, the highest prevalence of chronic HCV infections is found in people who inject(ed) drugs (PWID), first generation migrant populations, and men who have sex with men (MSM) [15], but migrant populations represent the largest group in terms of absolute numbers of chronic infections. The COVID-19 pandemic may differentially impact the detection of chronic HCV infections among these populations. The current study examined the impact of COVID-19 and its related measures on (trends in) the number of chronic HCV infections from 2019 to 2021, separate for each route of transmission (MSM, PWID and other/unknown).

## Methods

### Data collection

The Dutch Public Health Act states that recently acquired and chronic HCV is classified as a group B2 infectious disease that is mandatory to report to the local Public Health Service (PHS) [16]. The PHS reports these cases to the National Notifiable Disease Surveillance System (Osiris) of the National Institute for Public Health and the Environment (RIVM) through an electronic questionnaire that includes demographic and epidemiological information including self-reported most likely route of transmission. Before 2018 only recently acquired HCV infections were notifiable, but since 2019 mandatory reporting of chronic HCV infections was introduced [17], because of the availability of highly effective treatment and to better monitor the (characteristics of the) HCV epidemic in the Netherlands.

A chronic HCV infection or infection with unknown duration is defined in the national guidelines of the RIVM as a first detection of HCV RNA and/or HCV core-antigen where the infection cannot be classified as a recently acquired infection or re-infection [15]. These infections are reported as ‘chronic/unknown’ (further described as ‘chronic’ under the assumption that the majority of the unknows is chronic). We included all chronic HCV cases, reported through Osiris, from January 2019-December 2021. For the time trend analysis per month, we used the date of diagnosis. For 79 cases (5.1%), date of diagnosis was missing, so we used date of reporting to the RIVM.

### Data analysis

Annual rates of newly reported cases of chronic HCV infection per 100,000 population and 95% confidence intervals (CI) were calculated [18]. We compared 2019 with 2020 and 2021 by using an unconditional maximum likelihood estimation (Wald) (R-package epitools) to calculate p-values.

Descriptive statistics were used to summarize annual numbers by age, sex, most likely route of transmission, HIV status, reporting PHS region, and country of birth. Also, we calculated proportional changes in the numbers of reported cases between the three calendar years of diagnoses. To determine if the reported route of transmission, sex, HIV status, reporting PHS region and country of birth correlated with the diagnosis year, we used Pearson’s chi square test (R-package stats). P-values lower than 0.05 were considered statistically significant. Statistical analyses were conducted using R 4.0.2 Statistical Software.

### Ethical approval

This study was conducted in accordance with relevant guidelines and regulations. Standard surveillance procedures were used to collect data regarding cases that were pseudonymized, as approved by the Dutch National Institute of Public Health. The need for ethical approval and informed consent was waived, as the collection of data complies with the exceptions for not asking informed consent as formulated under article six in the Dutch Implementation Act General Data Protection Regulation (GDPR).

## Results

Between 2019 and 2021, a total number of 1,521 cases of chronic HCV infections were reported to the RIVM. The rate per 100,000 population was significantly higher in 2019 (3.88; 95% CI 3.59–4.18) compared to 2020 (2.24; 95%CI 2.01–2.46, p < 0.001), and also compared to 2021 (2.63; 95%CI 2.39–2.87, p < 0.001).

**Fig. 1:**
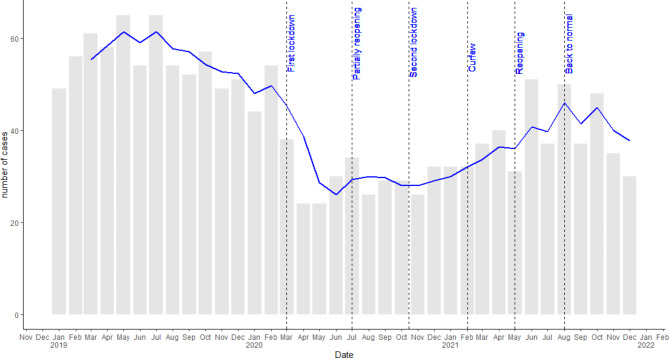
Chronic Hepatitis C virus cases per month including the timeline of COVID-19 measures in the Netherlands, 2019–2021

An overall decline of 41.9% in reported chronic cases was observed from 2019 (n = 671) to 2020 (n = 390), with the sharpest decline at the start of the COVID-19 pandemic (March-April 2020), when the first lockdown was implemented in the Netherlands (Fig. 1). An increase of 17.9% in reported cases was observed from 2020 to 2021 (n = 460) (Table 1; Fig. 1).

**Table 1:** Most likely route of transmission of chronic Hepatitis C virus in the Netherlands, 2019–2021

	Total		2019		2020		2021		2020 vs. 2019		2021 vs. 2020		2021 vs. 2019	
	n	%	n	%	n	%	n	%	%	p-value	%	p-value	%	p-value
PWID	498	32.7	218	32.5	131	33.6	149	32.4	+ 3.6	0.629	-9.0	0.237	-5.78	0.403
Needle-stick/bite/blood incident	121	8.0	48	7.2	26	6.7	47	10.2	-6.7	0.763	+ 44.6	0.101	+ 35.0	0.113
MSM	67	4.4	45	6.7	11	2.8	11	2.4	-57.9	**0.005**	-20.0	0.592	-66.3	**> 0.001**
Other risks (e.g. maternal, heterosexual risk, unknown sexual risk)	204	13.4	75	11.2	56	14.4	73	15.9	+ 28.7	0.106	+ 4.3	0.784	+ 34.2	**0.042**
Total without unknown route of transmission	890		386		224		280		-41.9		+ 25.0		-27.5	
Unknown route of transmission - Born in the Netherlands - Born abroad - Country of birth unknown	631 117 369 145	41.5	285 57 152 76	42.5	166 28 98 40	42.6	180 32 119 29	39.1	0.2 -15.7 + 10.7 -9.6	0.977 0.447 0.360 0.590	-8.1 + 5.4 + 12.0 -33.1	0.310 0.899 0.360 **0.036**	-7.9 -11.1 + 24.0 -39.6	0.262 0.345 0.213 **0.004**
Total	1,521		671		390		460		-41.9		+ 17.9		-37.0	

PWID: People who inject(ed) drugs, MSM: Men who have sex with men, HIV: Human Immunodeficiency Virus

The overall median age at diagnosis was 52 years [IQR 40–61], and 71.8% were male (n = 1092) (Table 2). 19% of the cases were reported by the Amsterdam Public Health (PHS) region (n = 289), and most were born outside the Netherlands (n = 855, 56.2%). Foreign-born countries most often reported were Poland (n = 133), Russia (n = 48) and Latvia (n = 48). Of cases, 5.5% was co-infected with HIV, but HIV status was unknown for more than half of reported cases (n = 861, 56.6%).

**Table 2:** Demographic characteristics of chronic Hepatitis C virus cases in the Netherlands, 2019–2021

	2019		2020		2021		Total	
	n	%	n	%	n	%	n	%
Total	671		390		460		1,521	
Sex - Male - Female - Unknown	486 185 0	72.4 27.6 0.0	282 108 0	72.3 27.7 0.0	324 135 1	70.4 29.3 0.0	1092 428 1	71.8 28.1 0.0
Age (in years) - Median - Min - Max	51 19 90		54 10 84		52 4 88		52 4 90	
PHS region - Amsterdam - Other	132 539	19.7 80.3	74 316	19.0 81.0	83 377	18.0 82.0	289 1232	19.0 81.0
Country of birth - The Netherlands - Other countries - Unknown	199 354 118	29.7 52.8 17.6	104 222 64	26.7 56.9 16.4	133 279 48	28.9 60.7 10.4	436 855 230	28.7 56.2 15.1
HIV status - Positive - Negative - Unknown	41 251 379	6.1 37.4 56.5	16 134 240	4.1 34.4 61.5	27 191 242	5.9 41.5 52.6	84 576 861	5.5 37.9 56.6

PHS: Local Public Health Service; HIV: Human Immunodeficiency Virus

For a large proportion of cases with chronic HCV infection the transmission route was unknown (631/1,521, 41.5%)(Table 2). Of the (self-)reported most likely transmission route, injecting drug use represented the majority of the cases (498/890, 56.0%), followed by needle-stick/bite/blood incident transmission risk (121/890, 13.6%), MSM transmission risk (67/890, 7.5%), and other risks (204/890, 22.9%) (Table 2)

Sex, median age, PHS region where HCV was reported, and country of birth did not statistically differ over the years (data not shown). Proportions of cases with HIV status changed between 2019, 2020 and 2021, however not statistically significant as the percentage of missing data was higher in 2020 when compared to 2019 and 2021 (56.5% versus 61.5% and 52.6%, respectively)(Table 2).

Of all documented routes of transmission, MSM showed the largest proportional decline in the reported number of chronic HCV infections over time: -57.9% from 2019 to 2020 (p = 0.005), and a further proportional decrease by 20.0% from 2020 to 2021, although not significant (p = 0.592)(Table 1). Needle-stick/bite/blood incident transmission did not significantly decline from 2019 to 2020 (p = 0.763), however increased by 44.6% from 2020 to 2021 although not statistically significantly (p = 0.101). For all remaining transmission routes the number of cases did not significantly differ between 2019 and 2020, and between 2020 and 2021. However, the group ‘other risks’ showed an overall significant increase of 34.2% between 2019 and 2021 (p = 0.004)(Table 1).

## Discussion

We explored the impact of COVID-19 measures on the reporting of chronic HCV infections in the Netherlands. A decline of 41.9% was observed from 2019 to 2020, followed by a partial recovery of 17.9% in the subsequent year. The decline was most pronounced for MSM, and no rebound was seen in cases among MSM in 2021, although numbers in this group were small and should be interpreted with caution.

As the overall trend in reported chronic HCV cases largely followed the trend of the COVID-19 restrictions, a reduction in HCV screening due to an overloaded healthcare system, along with patients’ reluctance to visit health services with health complaints during the pandemic, could explain these parallel trends. According to the Dutch Patient Federation, in April 2020 only 11% of all hospital consultations continued as usual [19]. Also Sexual Health Centers (SHC) showed a strong decline of 30% in the number of visits to the SHC for consultations for sexually transmitted infections between 2019 and 2020, with the largest decline in April and May 2020 [20]. This could have affected the detection of HCV infections among MSM, although MSM on daily pre-exposure prophylaxis (PrEP) continued to return for care during the COVID pandemic [21]. While numbers of HCV diagnoses at SHC are small, and no distinction between infectious, cleared and treated HCV was available before 2020, the number of HCV diagnoses clearly declined among MSM from 2019 (n = 56) to 2020 (n = 35) [20].

Similar decreases in HCV health care availability were seen in Germany [22], England [23], the United States [24, 25], and in a recent survey conducted by the European Association for the study of the Liver [26]. Besides patients not being able to access testing facilities, delayed health care seeking behaviour and changes in risk behavior were observed during the pandemic. Nab et al. investigated motivations for delayed emergency department visits in the Netherlands [27]. Patients described reasons as fear of COVID-19 contamination, not wanting to burden health professionals, and perceiving their own complaints as less urgent relative to COVID-19 patients. Individuals might have postponed or cancelled regular healthcare check-ups and screening, which may have resulted in a lower detection of chronic HCV infections.

Besides a decrease in HCV health care availability, the decline in reported chronic HCV might be related to a decline in behaviour associated with HCV infection. For example, in the cohort study of van Bilsen et al., 38% of participating MSM reported a reduction in the number of casual sex partners during COVID-19 restrictions [28]. Furthermore, the trend of the reported number of diagnosed recently acquired HCV infections in the Netherlands showed a similar decline as the chronic HCV infections, with a decrease of 30% between 2020 and 2021 [29]. However, we expect that a decline in recently acquired HCV infections, due to reduced sexual risks during the pandemic, had little impact on the current rates of chronic HCV. The detection of a chronic HCV infection can take decades, as most patients do not immediately experience symptoms [30, 31], and seek healthcare by themselves. A decline in HCV-screening and -treatment, therefore, could subsequently lead to an increase in HCV associated morbidity and mortality, especially if missed cases during that period do not enter care in a later phase [12]. Hence, it is promising that numbers of diagnosed HCV infections increased again in 2021, likely as a result of the recovery of the healthcare system. However, as HCV infections are typically not diagnosed as a result of cirrhosis-related complications but as a result of additional testing upon finding elevated liver values [12], it remains unclear whether missed HCV patients due to COVID-19 restrictions in 2020 will re-enter the healthcare system in the short term.

### Limitations

We used routinely collected surveillance data to guide policy making, which includes self-reported data on transmissions route, which is prone to recall bias. Also, the dataset used included only one year prior to the COVID-19 pandemic, as chronic HCV is mandatory to report from 2019 onwards. Furthermore, we cannot exclude that in our reference year 2019, the start of the surveillance of chronic HCV cases, could have biased the trend towards higher numbers in 2019. For example, public health services might have reported cases identified before 2019 as newly found cases or physicians might have made more effort to find and diagnose chronic HCV cases due to a renewed awareness for chronic HCV, thus resulting in an overestimation of the COVID-19 effect on the incidence of chronic HCV. However, when comparing our data with all diagnosed HCV infections collected through sentinel surveillance among a nationwide network of designated laboratories, no significant change in the percentage of infections reported is present for 2019 and 2020. In 2019, 357 cases (53%) were reported in the sentinel surveillance and 671 in our dataset, and in 2020 this was 211 and 390 respectively (54%) [32]. This suggests that chronic HCV diagnosis numbers were relatively stable in the years preceding the pandemic, and that 2019 is a valid reference year.

Another limitation is the large proportion (41.5%) of missing data on transmission routes. The majority of chronic HCV infections occurs in the so-called ‘difficult-to-serve’ populations including people with a migration background and PWID. Professionals may have encountered cultural and language barriers during source finding interviews. Also, recall bias is likely as patients could be infected years or even decades ago, and might not remember the most likely route of transmission. Therefore, it remains difficult to explain the observed trends among the group with un unknown route of transmission.

## Conclusions

In conclusion, we observed a substantial impact of the COVID-19 pandemic on reported chronic HCV infections in the Netherlands, especially for MSM-related. This was also the first evaluation of the newly implemented mandatory reporting of chronic HCV in the Netherlands, and trends in chronic HCV cases notified should be closely monitored over the following years. Based on our results, we have updated the surveillance questionnaire for chronic HCV cases reporting by including additional information on the applicant of the diagnostic test to gain more insight in locations of HCV testing and potentially also on transmission risks. With further research we get more insight into the impact of access to healthcare, health seeking behaviour, and (sexual) transmission risks during the pandemic on reported chronic HCV infections.

## Data Availability

The datasets generated and/or analysed during the current study are available from the corresponding author on reasonable request.

## List of abbreviations

CI: Confidence intervals
DAA: Direct-acting antivirals
HCV: Hepatitis C Virus
HIV: Human immunodeficiency virus
MSM: Men who have sex with men
PHS: Public Health Service
PWID: People who inject(ed) drugs
RIVM: Dutch National Institute for Public Health and the Environment
SHC: Sexual Health Centers
